# Robust diagnostic classification via Q-learning

**DOI:** 10.1038/s41598-021-90000-4

**Published:** 2021-06-03

**Authors:** Victor Ardulov, Victor R. Martinez, Krishna Somandepalli, Shuting Zheng, Emma Salzman, Catherine Lord, Somer Bishop, Shrikanth Narayanan

**Affiliations:** 1 University of Southern California, Los Angeles, USA; 2 University of California San Francisco, San Francisco, USA; 3 University of California Los Angeles, Los Angeles, USA

**Keywords:** Health care, Diagnosis

## Abstract

Machine learning (ML) models have demonstrated the power of utilizing clinical instruments to provide tools for domain experts in gaining additional insights toward complex clinical diagnoses. In this context these tools desire two additional properties: *interpretability*, being able to audit and understand the decision function, and *robustness*, being able to assign the correct label in spite of missing or noisy inputs. This work formulates diagnostic classification as a decision-making process and utilizes *Q*-learning to build classifiers that meet the aforementioned desired criteria. As an exemplary task, we simulate the process of differentiating Autism Spectrum Disorder from Attention Deficit-Hyperactivity Disorder in verbal school aged children. This application highlights how reinforcement learning frameworks can be utilized to train more robust classifiers by jointly learning to maximize diagnostic accuracy while minimizing the amount of information required.

## Introduction

Clinical diagnoses represent a time-sensitive, and high stakes domain in which experts are called upon to seek and assess presenting symptoms, test results, and histories against the plethora of clinical conditions to characterize, and categorize, the patient’s condition. Due to the wide variety in clinical conditions and presentation, and the complex relationships between them, data-driven models have become increasingly adopted to coalesce these multimodal streams of information. A number of works have demonstrated the promise of machine learning (ML) models in these domains, addressing many clinical challenges such as patient privacy and medical data-mining^1–4^. However, many of the computational approaches operate under the assumption that the source of data for these ML models are all available at the same time. Clinical practice, especially in complex decision making, typically involves a progressive multi-step approach, starting with the collection of a history and symptoms on admission. With this information a practitioner can form initial hypotheses which are then supported or rejected by further tests and data gathering which can then either be followed up on, or used to establish a diagnosis. Following this it seems that a natural extension to the adoption of ML models is to reformulate the diagnostic procedure as a decision process, rather than a detection and classification problem in which an intelligent agent (the practitioner) is taking actions that have a cost but progressively reveal information about the underlying condition they are attempting to characterize.

To demonstrate the effectiveness of this task formulation, we will ground our work in addressing challenges associated with determining a child’s risk for a neurodevelopmental disorder. In particular, with the latest Diagnostic and Statistical Manual for Mental Disorders, Fifth Edition (DSM-5) introducing joint diagnosis of Autism Spectrum Disorder (ASD) and Attention Deficit-Hyperactivity Disorder (ADHD), clinicians are posed with a challenge differentiating between the two, despite having overlapping presentations. Specifically, certain behaviors and subsequent social skills presented in children with ADHD have a well documented overlap with those associated with ASD^5–9^. This results in children being characterized as being at-risk for conditions they may not have, potentially delaying the correct evaluation, diagnosis, and subsequent interventions.

Within the domain of ASD diagnosis, prior work has evaluated the sensitivity of ASD diagnostic instruments differentiating the two conditions in children known to have non-comorbid ADHD and those with a known ASD diagnosis^10,11^. Currently, when a child is considered at-risk for ASD they and their care-givers are routed through a series of extensive diagnostic procedures composed of observational items, both care-giver reported and clinician observed. These items rate the behaviors and developmental abilities exhibited by a child. Diagnostic algorithms are then used to aggregate the scores to inform clinicians and allow them to more methodically determine diagnoses or to triage children to further appropriate screening. The rigor and thoroughness of these instruments, while time-intensive, are designed with the consideration of the high stakes and consequences of misdiagnosis.

Machine learning (ML) models have been presented as analytical tools in order to develop a stronger understanding for the differentiating factors of ASD and ADHD from the data available. However due to the significance of the domain, it is important that diagnostic models adhere to two critical characteristics: The first, *robustness*, aims to promotes the ability of the model to predict effectively with less information or with missing and noisy information. The second characteristic, *interpretability*, promotes a traceable decision path, enabling a human readable decision flow that determines how a model came to a conclusion for a particular data input.

Prior work on clinical diagnostic ML approaches have largely utilized decision tree or forest based models in order to select predictive and reduced sets of items^12–14^. While capable of generating decision paths which accommodate the second desiderata presented above, concerns over the choice of data and the generalization of these results underscore the difficulty of validating the *robustness* of these models^15,16^.

By selecting a more appropriate dataset and more thoroughly outlining the notion of a robust classifier, our work presents a novel application of a reinforcement learning (RL) framework towards constructing a simultaneously robust and interpretable classifier. The work demonstrates the limits of tree and forest based models illustrating their sensitivity to data omission and corruption. The methodology outlines an approach to build adaptive strategies able to guide and recommend which item to inquire next as more information about the child becomes available. This reformulation establishes this diagnostic setting as a decision making process, for which an optimal policy can found through the policy optimization method known as *Q*-learning. Through simulation of diagnostic processes, the policy jointly maximizes diagnostic accuracy, while minimizing the number of items needed to assign a diagnosis, thus eliciting a policy that is adaptive to the available information and less dependant on any single feature to make a diagnosis. In the end the policy can be interrogated similarly and interpreted similarly to a tree based model, however the advantages in robustness become more apparent. This work highlights the advantages of this reformulation and presents an illustrative evaluation of *Q*-learning as applied to the ASD-ADHD diagnostic domain.

## Background

### Autism diagnostic interview—revised

ASD is a neurodevelopmental disorder (NDD) that primarily manifests itself in an individuals’ ability to conduct and regulate behaviors associated with social communications^17^. In an effort to more consistently assess children for ASD symptoms, a number of different instruments consisting of a variety of clinically-relevant items have been developed that enable diagnostic algorithms. While these instruments and associated algorithms are not used to diagnose, they are tools that the clinician can rely on in determining a child’s risk and need for further diagnostic observation. Many different instruments measure similar behaviors, but differ in who the observer is and how the observations are made, varying between parent/caregiver reported to direct clinician observation, leading to varying reliability and accuracy for certain observations.

**Figure 1:**
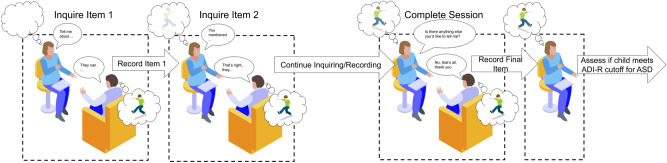
ADI-R administration process. The parent is interviewed by a clinician. The clinicians asks open-ended questions that are tied to an item and listens to the responses from a parent. Typically the clinician is listening and asking about specific examples of the child’s behavior in relation to the item at hand. The clinician records a rating based on the presented information and can leave notes to themselves. After the interview is complete the clinician uses their recorded ratings to complete the ADI-R algorithm computing whether the child meets the instrument’s cut-off thresholds for ASD.

One of those widely used instruments is the Autism Diagnostic Interview-Revised (ADI-R), which is a clinician led diagnostic interview, during which the child’s caregivers are asked a series of questions associated with evaluating the child’s communication abilities and social behaviors^18^. The clinician assesses responses to these questions to determine whether the child’s behavior fits within expected behavior given the child’s background. If through the course of interview the clinician can determine that the child should not be considered at-risk for ASD, either because they are typically developing or have a different NDD, it could prevent further unnecessary testing and can direct a child to a more appropriate diagnostic procedure or treatment option. A more thorough description of the interview process is described in Fig. 1.

Recent works evaluating the differentiation of ADHD and ASD in at-risk children have demonstrated the statistical sensitivity in identifying the symptoms and items which most significantly distinguish the two conditions^10,11^. These have evaluated different instruments, including ADI-R, in verbal, school-aged, clinically referred children. Similar studies have explored the use of machine learning (ML) models, specifically Decision Tree based models, to differentiate and identify specific features which have diagnostically relevant information^12,13,15,16^. Tree models are preferred for their interpretability however, our results demonstrate that they typically are poorer classifiers than those learned by *Q*-learning at accurately predicting in the case of corrupted data, either by omission or noise.

### Reinforcement learning

Reinforcement learning (RL) is a paradigm for identifying strategies or algorithms that solve complex, often sequential, decision making problems, optimizing the total reward gained over the length of an interaction^19^. By formulating a problem as sequence of actions made by an agent which is rewarded or penalized, it is possible to learn long-term strategies that can learn complex behaviors such as local sacrifices of reward for long-term pay-out.

This realm is closely related to the domains of game-theory and optimal control theory^20^ in which rewards and errors are used to define the utility of a policy. In both domains the definition of a policy can be generalized as a function $\pi :S \rightarrow A$ where *S* represents a state-space of an agent or system being controlled and *A* represents an action space. A reward function $r:S \rightarrow \mathbb {R}$ is a function that informs the agent about the utility of reaching a specific state.

Given a discrete state-space and discrete action-space the decision making process is a graph with nodes and edges representing states and actions respectively. There are three types of states: initial, passive, and terminal. An initial state is one that the agent can find themselves initialized with, and a terminal state is one that has no outgoing edges thus terminating the decision-making process. A path $p = [s_0, s_1, \ldots , s_{n-1}, s_n]$, represents the order in which the states are visited, a list beginning with the initial state $s_0$, followed by passive states $s_1, \ldots , s_{n-1}$, finishing with the terminal $s_n$, where some state $s_{j+1}$ was reached from $s_{j}$ by taking some action $a_j$.

An optimal policy $\pi ^\star$ is one where the sequence of states visited defined the actions selected according to the policy compose a path $p_\star$ such that:

$$\begin{aligned} \sum _{s_i \in p_\star } r(s_i) = \max _{p \in P}\big (\sum _{s_j \in p} r(s_j) \big ) \end{aligned}$$

When state transitions are stochastic, the problem is known as a Markov Decision Process (MDP), implying that performing the same action in a particular state may not always yield the same transition. If the transition probabilities to a MDP are not known apriori, a common approach to find the optimal policy is to learn through simulation. One such learning method, *Q*-learning, attempts to approximate the *Q*-function mapping state-action pairs onto a real value representing the “quality”.

The quality of taking an action $a_j$ given non-terminal state $s_j$ is the reward received at the current state plus the expected sum of $\gamma$-discounted future rewards received from all states following action $a_j$. More explicitly:

$$\begin{aligned} Q(s_{j}, a_j) = r(s_{j}) + \mathbb {E}[\gamma r(s_{j+1}) + \gamma ^2 r(s_{j+2}) + \ldots + \gamma ^n r(s_{n})] = r(s_{j}) + \gamma \max _{a' \in A} Q(s_{j+1}, a') \end{aligned}$$

The *Q*-learning paradigm and its variations have demonstrated recent success, achieving super-human performance in complex games, namely playing Atari games when using deep architectures^21^ and defeating the reigning human champion of Go when paired with Monte-Carlo Tree Search^22^. This work builds to adapt these principles to leverage the long-term planning capabilities in the differentiation of NDDs in children based on behaviors that are observed by their guardians. The key to this formulation is to consider the diagnostic instrument as an MDP in which the clinician acts as the agent of a policy which suggests the items to follow-up on to reach a desired end state where their prediction, based on observed responses to actions, matches the underlying true label for the child.

## Data

Querying a dataset of clinically referred children as being at-risk for ASD, an initial sample of 119 children with best clinical estimates of ADHD were assigned by experts. Then the Full Scale IQ (FSIQ) and age of referral were used to restrict a query of children with best clinical estimates of ASD. These restrictions allow the analysis to focus on groups of children that would present most similarly in terms of developmental ability. When FSIQ was not available a Verbal IQ (VIQ) was used to determine whether a child with ASD belonged in the sample. The final data sample consisted of 463 children with 344 children with clinical estimates of ASD.

**Figure 2:**
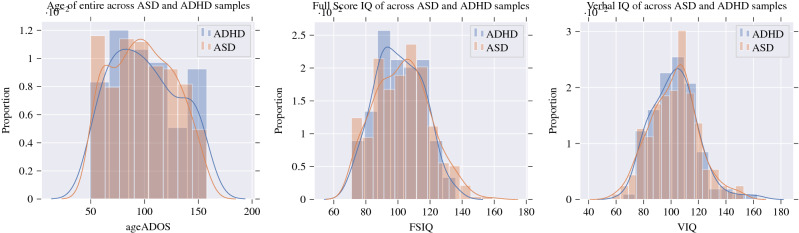
Distribution of demographic information: age, FSIQ and VIQ across different diagnostic conditions.

Figure 2 visualizes the distributions by clinical estimates and Table 1 shows the averages, standard deviations and *p* values associated with evaluating Student’s *t*-test. The yielded *p* values suggest that we cannot reject the null hypothesis that the distributions are significantly different.

**Table 1:** Demographic distributions.

Variable	Average	*t*-test *p* value
Age	99.06 ($\sigma = \pm 29.34)$	0.5639
FSIQ	102.15 ($\sigma = \pm 16.54)$	0.2615
VIQ	102.21 ($\sigma = \pm 17.15)$	0.9396

The *p* values for t-tests suggest that we cannot reject the null hypothesis that they are from different populations according to these features.

Further still, although not used in sample selection similar distribution of sex were observed—ASD contained 80% male to 20% female while ADHD was 74% male to 26% so as this is not conflated in the diagnostic features, consistent with globally observed statistics across clinical referrals for ASD^23^.

Our experiments focus on the use of ADI-R items to predict the correct ASD/ADHD clinical estimate. Each item is scored on a scale [0, 2] where 0 implies typical behavior and 2 represents significantly atypical behavior. A subset of the ADI-R items are chosen using Student’s *t*-test to identify the 10 items that are maximally differentiated across the two diagnostic conditions, comparing each item’s capacity to distinguish ASD from ADHD. This was done to account for the way in which the states will be encoded in the policy and the exponential growth of the state-space to accommodate for each new item. This limitation is described in more detail in “Q-learning” section. Table 2 explicitly states the 10 items used as well as the relative *t*-statistic.

**Table 2:** Items used, their descriptions and the Student’s *t*-test statistic.

Item	Description	*t*-test statistic
ADI_35	Current:reciprocal conversation	9.2263
ADI_34	Current:social vocalization/chat	7.1570
ADI_68	Current:circumscribed interests	6.5214
ADI_51	Current:social smiling	6.3941
ADI_33	Current:stereotyped utterances and delayed ech...	6.3521
ADI_42	Current:pointing to express interest	6.2030
ADI_59	Current:appropriateness of social responses	6.1215
ADI_45	Current:conventional/instrumental gestures	5.7257
ADI_57	Current:range of facial expression used to com...	4.9405
ADI_72	Current:undue general sensitivity to noise	4.7258

All *p* values $< 1.06 \times 10^{-3}$ indicating significance with Bonferroni correction.

In order to evaluate the methods and models trained more effectively, our experiments utilize a 10-fold stratified cross-validation (CV). This method allow us to validate that the results of model performance were not sporadically produced by a lucky train-test split.

## Methods

### Baselines

In diagnostic modeling, it is key to interpret not only the output of the model, but the path to that decision^24^. For this reason prior work on autism diagnosis has explored largely the use of tree and forest based models. With these characteristics in mind, we outline a benchmark consisting of Decision Tree (DT) and Random Forest (RF) models that are trained on Stratified 10-fold Cross Validation (CV) to better approximate our performance on evaluation and expected generalization of the learned models.

Two types of benchmark models are trained: the first with no data-augmentation that learns from the original training samples as is and the second set of these models are trained using a batch of masked data. In this case the masking operation selects a number of items and drops them from the input. In order to allow these models to still make predictions the missing data is interpolated with the median value for the missing column from the training split. This configuration allows us to synthesize more examples for training the model. Furthermore, these new data can potentially introduce counterfactual examples to those observed in the original sample requiring the model to learn a more robust classifier which is less likely to fail with imperfect inputs.

In order to confirm whether the models are learning significant representations of the data, a bootstrapped baseline is generated by randomly sampling for a label from the same distribution as the original dataset. Simulating this process 10000 times produces a distribution of expected performance to compare against. The 95th-percentile of the F1 distribution, 0.4428, is taken for the comparisons. If a model’s average CV F1-score is above this threshold then it implies that the model generally performs significantly better than random guessing thus suggesting that the relationships learned by the model is likely significant.

### Q-learning

The implementation of our policy relies on two major elements: The first is a Naive Bayes classifier (*G*) that is used to make the final prediction and the policy $\pi$ which utilizes a table to discretely approximate the *Q*-function.

The formulation begins by reconsidering the ADI-R scores as observations made by an agent after taking an action. In particular, we consider an action as “asking” a question and receiving the score for that item as the observation. We use 0 to represent missing information and then shift the true severity score by adding one to represent the observed score. Now by evaluating the observation as 4-bit number, we can convert the observation into a state that can be used as key in the policy table to look up the *Q* values of each action when in that state.

The Naive Bayes classifier is trained on a masked set of training inputs, similar to those outlined in the ML model baselines. This function *G*, maps observations to a 2-dimensional vector, each entry represent the classifiers’ assigned likelihood of belonging to each possible class. This function is used for two things: First, it is used during training in order to assign an intermediate reward to the policy informing the policy when the model is steering the observation towards allowing the classifier to make the appropriate classification. Secondly, by adding the PREDICT action to the action-space, the policy feeds whatever state it is currently in to the classifier and a final reward is given depending on whether or not the classifier produced the correct predicted label. Equations 1 and 2 outline the local and final rewards used to train the policy $\pi$ more explicitly.

1 $$\begin{aligned} r_{local}(s, G, y)= & {} \beta \big (2\big (G(s)[y] - \frac{1}{2}\big )\big ) \end{aligned}$$

2 $$\begin{aligned} r_{final}(s, G, y, h, l)= & {} {\left\{ \begin{array}{ll} \beta + C\big (1 - \frac{h}{l}\big ) &{} y = \arg \max \big (G(s)\big )\\ -1 &{} else \end{array}\right. } \end{aligned}$$

During each iteration we simulate the process of starting with no information and allow the agent follow its current policy, observing the reward and state transition and updating their *Q*-function at each interaction. Additionally, during training a parameter $\epsilon \in [0, 1)$ is used as the exploration probability. This is the probability that during training the agent will choose a random action (explore) over following its current policy (exploit). For the purposes of these experiments an $\epsilon = 0.2$ is used. Further still, the discount parameter $\gamma$ is set to 0.99, and the learning rate $\alpha$ is set to 0.01. The policy cannot repeat actions, once one has already been taken. A more thorough process for training the policy can be found in Algorithm 1 in Appendix B.

Each fold trained the policy on the same set of training examples as those used in the ML models so that the comparisons across folds could account for splits in the data. Table 1 in Appendix A organizes all variables and functions referenced to compute the reward and prediction of the policy.

During training, the policy is evaluated on a withheld development set. While there is an upper threshold for how many iterations the policy can learn for, the reward received when evaluating on the development set is also used as an early stopping condition in the event the model stop improving its development reward for more than 30 iterations.

### Experiments

**Figure 3:**
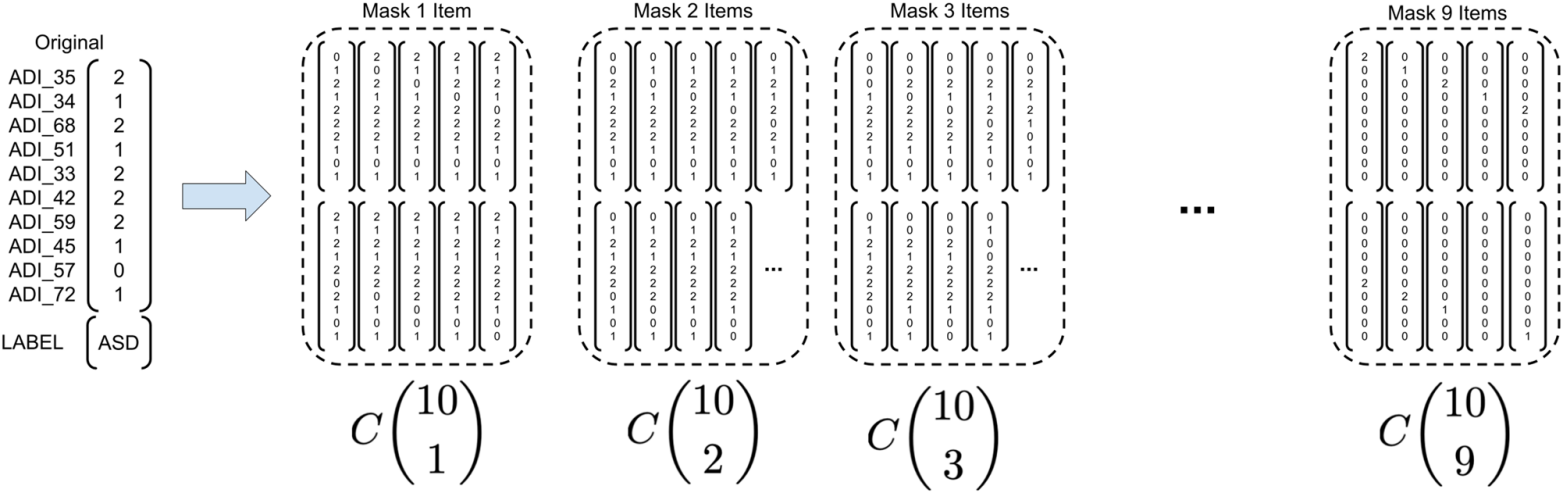
Process demonstrates how a single example is converted into masked examples. The 0s represent values that are unavailable to the classifier *a priori* and will be potentially imputed. The notation $$C\left( {\begin{array}{c}m\\ n\end{array}}\right) $$ (*m* choose *n*) represents the number of examples generated by masking *n* items.

The performance and robustness of the Decision Tree (DT) and Random Forest (RF) models and the reinforcement learning (RL) model, are evaluated through prediction of diagnostic labels in a 10-fold stratified cross validation. For each fold, 10 percent of the data is withheld from training and combinatorially masked. This means that for each sample in the test set a “corrupted” sample is created by omitting a subset of the features. For the DT models this omission is replaced with that item’s median value from the training split in the same manner that they were to train the robust models. For the RL missing data remains encoded as 0. This omission process produces every combination of possible mask as applied to the 10 items in any given sample. An example of the masking process on a single example can be seen in Fig. 3.

The experiment with masking demonstrates how the models perform with incomplete knowledge and allows us to determine whether they are learning robust representations. A more robust model should recover a correct diagnosis and perform better than the bootstrapped baseline. A classifier that is able to successfully predict despite missing data, demonstrates that the policy could generalize correctly to an instrument with fewer items.

## Results

Table 3 shows how the different models performed first on the completely available dataset, and then when information was missing. The table shows us that the DT is the only model that initially does not out perform the randomised baseline set forth in “Baselines” section. Notably, the learned policy out performs all of the ML models and is the only model to maintain a competitive F1 score even when 9 features are inaccessible.

**Table 3:** F1-Score initially when all data is available, subsequently when only one feature is available.

Items Masked	DT	RF	$\hbox {DT}_{\mathrm{robust}}$	$\hbox {RF}_{\mathrm{robust}}$	*Q*-learning ($\pi$)
0	0.4131	0.4753	0.5044	0.5064	**0.5620**
9	0.0307	0.0000	0.1211	0.1211	**0.4312**

**Figure 4:**
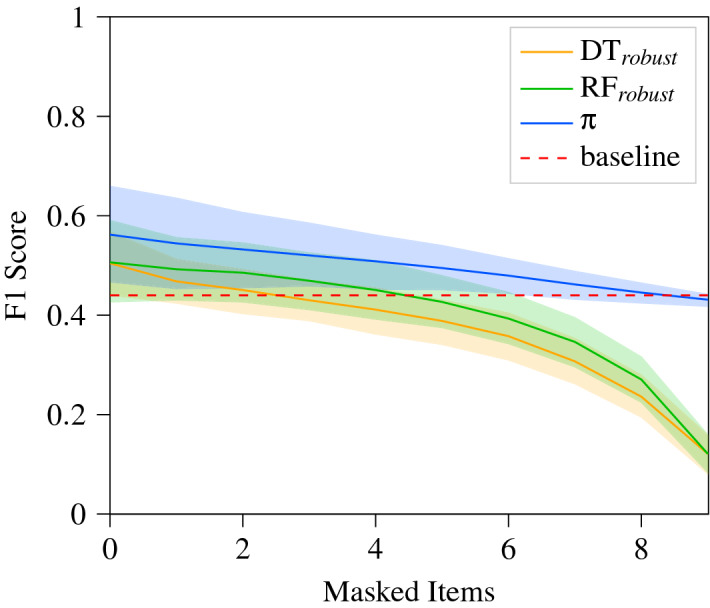
F1-Score degradation as more features are masked from the inputs.

These results suggest that the policy is learning to correlate each item independently with the diagnostic label while also learning the relationships between the features. These results can also be seen more gradually in Fig. 4 where the degradation of the ML models performance against the baseline is seen with higher granularity. The $\hbox {DT}_{\mathrm{robust}}$ and $\hbox {RF}_{\mathrm{robust}}$ degrade much more steeply than the policy $\pi$ suggesting that the underlying states and structures learned by the policy are more robust than the representations learned by the ML models.

### Policy

**Figure 5:**
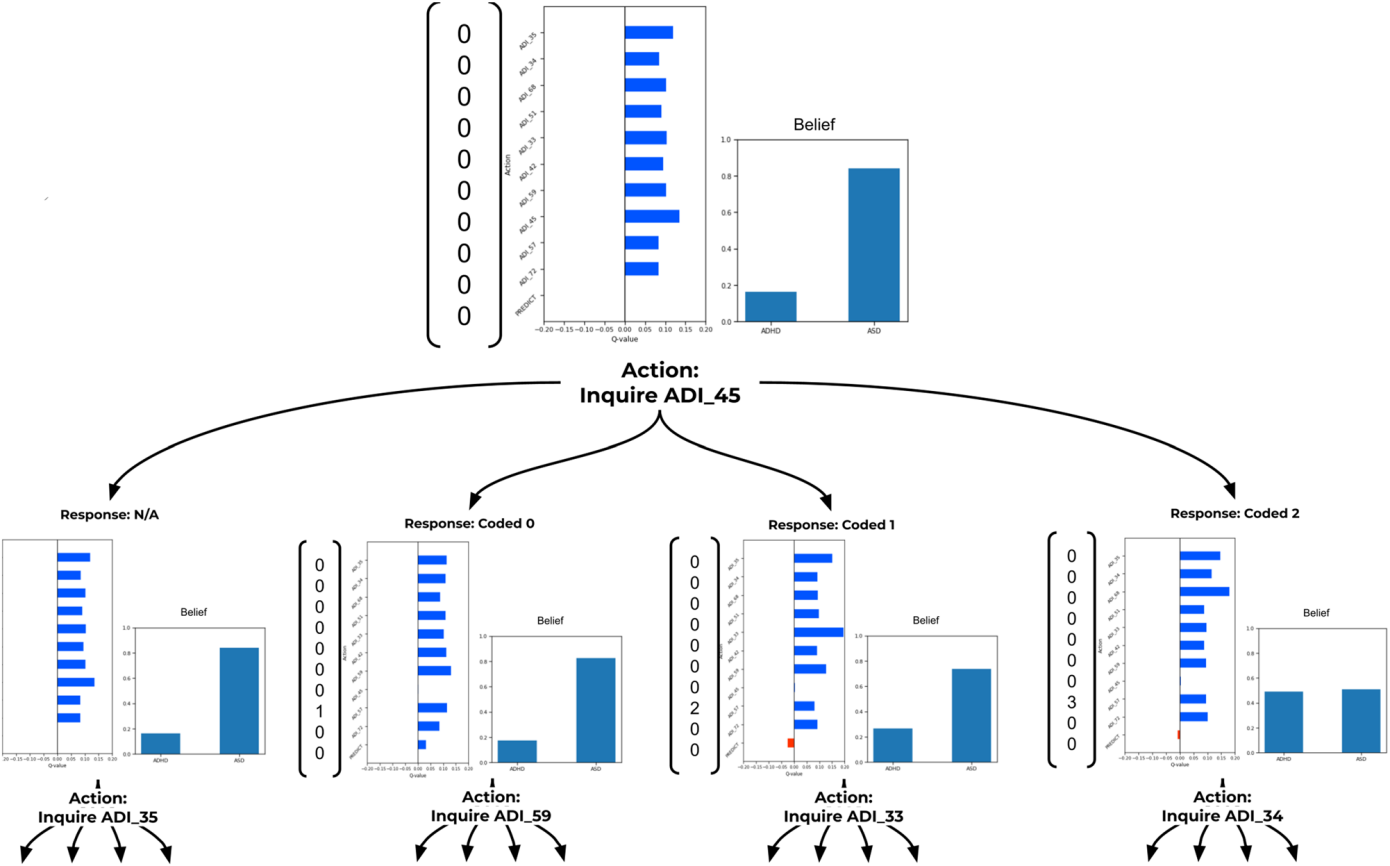
An example of how a policy updates with all possible responses from an inquiry. The top row captures the initial “empty” state of the policy, while the branch represent all of the possible state update that could occur depending on the observation made following the action taken. The column vector represents the state of the policy, or the items that the policy has information about so far. The horizontal bar chart captures the relative Q-value of each action (actions are equivalent to querying an item or making a prediction). As ADI_45 has the highest Q-value, it is the first item that is queried by the policy. The arrows capture possible responses, or observations, that the policy can have, which in turn are used to update the state. The verticle bar chart captures the current state’s predicted probabilities of ADHD and ASD respectively (*Belief*).

In line with our goal to maintain interpretability of the policy, Fig. 5 demonstrates how the learned policy for one of the folds adapts to responses depending on which response is returned. The figure shows how it is possible to trace back from a diagnosis, see what the diagnostic belief state is according to the Naive Bayes classifier trained for the RL model, and how in certain circumstances the policy learns to avoid predicting too early, when too little information is available.

Another way to better interrogate the representations learned by the ML models and the policy is to understand the feature importance for each of the models. For the policy, the feature importance can be thought of as the *Q*-value of any action given a state and is adaptive through out the session as seen in Fig. 5. To interpret a more global representation of feature importance is to look at the output of $Q(0, \cdot )$ as this is the priority of items to ask when no other information is presently available. Although it is important to note that in any state the policy may produce a different importance for future decisions.

**Figure 6:**
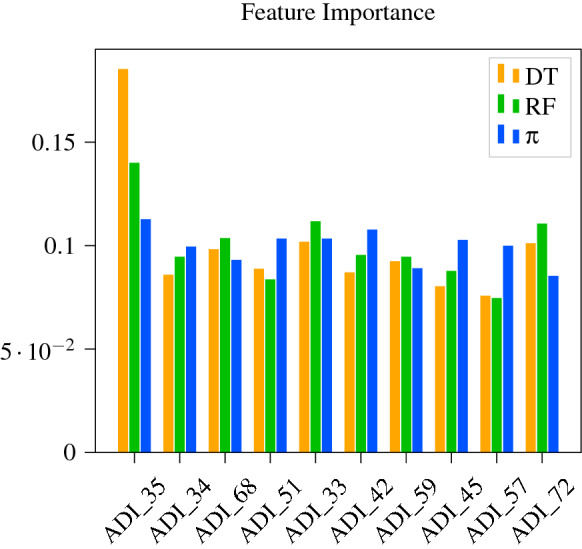
Importance of different items relative to each other according to different model types.

Figure 6 shows some of the underlying differences between the ML models and policy. Namely, it seems that the policy learns to more evenly consider the features than the tree based models. This is supported further by the observation that the variance and range of the feature importance for each of the classifiers shown in Table 4.

**Table 4:** Variance and range of feature importance for each of the classifiers.

Model	Variance	Range
$\hbox {DT}_{\mathrm{robust}}$	$8.8 \times 10^{-4}$	0.1089
$\hbox {RF}_{\mathrm{robust}}$	$2.7 \times 10^{-4}$	0.0635
$\pi$	$6.3 \times 10^{-5}$	0.0273

The difference in these is likely a byproduct of the exploration procedure allowing the model to learn better partial representations. Similarly, since the quality of an action is dependent on all future states and rewards the *Q*-function for a given state-action pair aggregates the expected results of future decisions.

## Conclusion

By training a policy through simulation, our models are able to learn best clinical estimates from missing or corrupted data. This method suggests that the models are not overly reliant on an individual item’s correlation with a target but may instead discover more nuanced representations that interpret the interaction between items. This enables an interpretation of possibly conflicting information from various items with the ability to follow up further into an interaction.

Furthermore the adaptive nature of this policy and the joint objective it optimizes implies that when the cases are clearly pointing towards one outcome the model will reach them with fewer interactions, suggesting the next most informative action in more complex scenarios. The result is a policy that considers all future possible outcomes before taking the next action and ingesting more information, thus it is possible to maximize its *robustness* while preserving its *interpretability*. An additional advantage is that while in the experiments the policy was allowed to choose and execute the next highest quality action according the reward, this method is also adaptable to spontaneous actions being taken. More concretely, the structure of the graph and algorithm of the policy will always recommend the next best action in terms of assigning the correct label from any state, regardless of how the agent reached that current state.

This work demonstrated that data-augmentation and *Q*-learning can be utilized in low-resource conditions to learn a more diagnostically robust representation of clinical data. By reformulating the process of diagnostic classification as an MDP, this work further outlines a computational framework for designing policies and utilizing the benefits of machine learning for adaptive diagnostic testing in uncertain data environments. The method for identifying and optimizing this policy, may be useful in designing future diagnostic methods which follow adaptive procedures by leveraging data available on current instruments. Specifically, by structuring future methods in a way that enables clinical practitioners to interact with a policy while they are administering diagnostic tests, a learned policy would be able to recommend prompts and items to explore that would be most powerful in solidifying a particular diagnosis while also accounting for potentially spontaneous observations that the clinician might make.

### Future work

The *Q*-table method for representing state-action value is limited in its ability to effectively scale as each new item introduces an exponential growth factor to table-size making it difficult to implement reasonably with too many possible states. For these reasons we suggest utilizing more sophisticated approach to representing the state. Deep reinforcement learning would enable the model to represent higher dimensional observation in lower dimension continuous state-space through their intrinsic embedding ability. This would have an additional advantage of potentially learning more effective strategies and meaningful structures that were unseen previously.

Further more, consider the case where there is a sequence of diagnostic procedures that can each be represented as an MDP. By using a continuous embedding to represent the states, these states can also be passed into “down-stream” policies as starting points for parallel diagnostic procedures. This could potentially produce multi-stage triage and lead to deeper understanding behavioral distinctions between different NDDs.

## Supplementary Information

Supplementary Information.
