# Supplementary material for: Robust diagnostic classification via Q-learning
Source: Sci Rep. 2021 Jun 3;11:11730. doi: 10.1038/s41598-021-90000-4 (PMC8175431; doi:10.1038/s41598-021-90000-4)
Supplement: Supplementary file 1 — Supplementary Information. [file 41598_2021_90000_MOESM1_ESM.pdf]

A Glossary of Variables and Terms

Variable	Bounds	Definition
i	$\in \mathscr{I}$	a specific item from the set of item $\mathscr{I}$ being analyzed
s	$\in S = \mathbb{Z}^+;\, s < 4^{\lvert\mathscr{I}\rvert}$	represents the state space used by the Q-table
a	$\in A = \mathbb{Z}^+;\, a < \lvert\mathscr{I}\rvert + 1$	actions to be taken by the agent
α	$\in \mathbb{R}^+$	learning rate for policy
γ	$\in [0,1)$	future reward discount
ε	$\in [0,1]$	exploration rate for training the policy
D		space for projected diagnostic predictions, dimensionality is equal to number of possible classes each component represents the probability that it belongs to a particular class
G	$G\colon S \to D$	Naive Bayes classifier that is used to evaluate intermediate states
β	$\in \mathbb{R}^+$	class bonus to alleviate class imbalance
C	$\in \mathbb{R}^+$	reward bonus for using less states, used to normalize against β
l	$\in \mathbb{Z}^+$	maximum allowable length of a session, default set to $\lvert A\rvert = \lvert\mathscr{I}\rvert + 1$
h	$\in \mathbb{Z}^+;\, h \le l$	length of the session on exit
Q	$Q\colon S \times A \to \mathbb{R}$	function mapping state-action pairs to real values representing the quality of taking an action given a particular state
π	$\pi\colon S \to A$	function that maps from state space into action space

Table 1. Definitions and limits on variables

B Q-Learning Algorithm

Algorithm 1 `Policy Training`$(X, Y, G, \mathscr{I}, T, \varepsilon, \alpha, \gamma)$

```
A ← 𝓘 ∪ {PREDICT}
for t ∈ [1, T] do
    shuffle(X)
    for j ∈ [1, |X|] do
        x ← X[j]
        y ← Y[j]
        s ← 0⃗_{1×|𝓘|}
        Â ← [1, |A|]
        h ← 0
        d ← 0
        while d ≠ 1 do
            u ∼ 𝒰([0, 1])
            if u ≤ ε then
                a ∼ 𝒰(Â)
            else
                a ← argmax_{a'∈Â} Q(s, a')
            end if
            Â ← Â \ {a}
            h ← h + 1
            if A[a] = PREDICT then
                p = 1[A[a] = P_ADHD]
                r ← r_final(p, y, h, |A|)
                Q(s, a) ← Q(s, a) + α(r − Q(s, a))
                d ← 1
            else
                s' ← s
                s'[a] ← x[a]
                r ← r_local(s, G, y)
                Q(s, a) ← Q(s, a) + α((r + γ max_{a'∈Â} Q(s', a')) − Q(s, a))
                s ← s'
            end if
        end while
    end for
end for
```

In Algorithm 1, $\mathscr{U}(\cdot)$ refers to a uniform density probability over a set which is being sampled ($\sim$).
